# E-mental health special issue

**DOI:** 10.1177/2055207616676792

**Published:** 2016-11-22

**Authors:** John Powell

**Affiliations:** University of Oxford, Oxford, UK

Mental health research has been at the forefront of digital health studies over the last two decades, and so it is fitting that the first special issue of *Digital Health* is devoted to papers in the field of e-mental health. Researchers, practitioners, and people with mental health problems themselves, have sought to harness digital tools to deliver benefits, including interactive digital interventions to treat or prevent problems, online information and education, applications and tools to monitor symptoms and provide feedback, and communities that provide peer support for a range of mental health conditions. Key current issues for researchers seeking to investigate the benefits of digital mental health tools include the extent to which interventions and support can be automated and remain effective and acceptable; the low levels of engagement and use found with many digital tools, together with high levels of attrition in intervention studies; the potential mental health benefit of social media interactions; and the huge explosion in mental health apps available via smartphones and how health services should evaluate and harness these.

At the same time, some aspects of our digitally enhanced lives may have negative impacts on our mental health: there are concerns about self-harm and suicide communities and pro-anorexia discussion groups, and there are issues of internet addiction and harmful use. It is now 16 years since Robert Kraut and colleagues wrote their seminal paper describing ‘the Internet Paradox’, subtitled ‘A social technology that reduces social involvement and psychological well-being’.^1^ While their later work provided more nuanced conclusions (that social outcomes are influenced by the personality of the user and the nature of the internet use),^2^ the point was made that researchers need to retain a critical perspective on the harms as well as the benefits in this area.

One area of particular current interest to policymakers is the extent to which low-cost, highly scalable, and often low-intensity interventions may be useful tools to deliver mental health promotion to groups at risk of mental health problems, thus reducing the burden on health services and potentially reducing later morbidity. In this special issue, two studies investigate whether new digital tools can be harnessed in different settings to promote mental health and support psychological wellbeing. Naslund and colleagues explore whether a Facebook group can offer a feasible and acceptable forum for health promotion among people with serious mental illness^3^ and Touloumakos and colleagues examine the conceptual and practical value of a personalized intervention for students in higher education facing mild to moderate psychological problems.^4^ A study by Stiles-Shields and colleagues provides a characterization of the multiple design elements used in cognitive behavioural therapy-informed interventions designed to prevent or treat depression or anxiety in young people.^5^

The other three papers in this issue explore different aspects of the e-mental health agenda. The area of online mental health information and support seeking is covered by Montagni and colleagues, who report on mental health-related internet use by university students and their level of trust in online sources.^6^ With smartphone ownership in the UK and the US now at around 70% of the population, the potential clinical benefit of mobile apps for mental illness is addressed by Corden and colleagues, who investigate the extent to which a mobile app for people with depression can improve medication adherence and depressive symptoms.^7^ Finally, the issue of digital harm is investigated by Zinoviev and colleagues who examine the self-declared interests of people who participate in non-suicidal self-injury communities online, providing insights for future work in this area.^8^

As always, we welcome your views and invite further contributions on the topic of e-mental health to the journal.
